# ACE2 and Apelin-13: Biomarkers with a Prognostic Value in Congestive Heart Failure

**DOI:** 10.1155/2021/5569410

**Published:** 2021-06-22

**Authors:** Cerasela Mihaela Goidescu, Roxana Mihaela Chiorescu, Mocan-Hognogi Larisa Diana, Mihaela Mocan, Mirela Anca Stoia, Florin Petru Anton, Anca Daniela Farcaş

**Affiliations:** ^1^Department of Internal Medicine, “Iuliu Hatieganu” University of Medicine and Pharmacy, Cluj-Napoca, Romania; ^2^Military Emergency Hospital “C. Papilian”, Cluj-Napoca, Romania; ^3^Department of Internal Medicine, Emergency Clinical County Hospital, Cluj-Napoca, Romania; ^4^Department of Cardiology, Emergency Clinical County Hospital, Cluj-Napoca, Romania

## Abstract

The progression of heart failure is the result of the interaction of several pathogenetic processes that involve the activation of biomarkers belonging to the renin angiotensin aldosterone system (RAAS), to its counterregulatory mechanisms, to the sympathetic nervous system and inflammation, and to oxidative stress. This study is aimed at determining the prognostic role of biomarkers in the evolution of patients with heart failure. These biomarkers are representative of different pathogenetic pathways involved in the progression of heart failure and the possible interrelationships between them and heart remodelling. *Method*. This is a progressive observational study on 53 hospitalized patients with low ejection fraction heart failure, who were followed up for 12 months. The aetiology of heart failure was ischemic heart disease and dilated cardiomyopathy. The patients were clinically and biochemically evaluated by EKG (echocardiography) on admission and at 6 and 12 months. The biomarkers included in the present study were angiotensin-converting enzyme type 2 (ACE2), apelin-13, NT-proBNP (biomarkers involved in the counterregulation of RAAS), interleukin 17 (IL-17), hsCRP (inflammatory biomarkers), and urinary 8-iso-PGF2*α* (oxidative stress biomarker). The evolution was considered unfavourable if the patients presented complications during hospitalization, were readmitted for decompensated heart failure, or died. *Results*. From the study group, 14 patients (24.52%) presented an unfavourable clinical evolution. The biomarkers that were associated with the evolution of patients during hospitalization were ACE2, apelin-13, NT-proBNP, and hsCRP. Multivariate logistic regression analysis identified ACE2 and apelin-13 as independent, predictive biomarkers for the unfavourable evolution of patients over the study period. Values of ACE2 above 4000.75 pg/mL and of apelin-13 less than 402.5 pg/mL were associated with an unfavourable evolution (poor clinical outcomes). *Conclusion*. The serum values of ACE2 and apelin-13 correlate with the unfavourable evolution of patients with reduced ejection fraction heart failure.

## 1. Introduction

In patients with heart failure, congestion is the dominant clinical profile and the main pathophysiological mechanism that causes organic dysfunction [1].

The therapeutic reduction of the congestion leads to improved organ function and improved patient prognosis [1].

The identification of blood and urine biomarkers, which are closely linked to the functional damage of the organs affected in congestive heart failure, is important for a better stratification of the prognosis of patients and for the development of new therapeutic methods [2, 3].

The renin angiotensin aldosterone system (RAAS) is a key pathophysiological mechanism in the development and progression of heart failure. RAAS is activated together with the sympathetic system in patients with heart failure as a compensatory mechanism to decrease cardiac output. It contributes to maintaining cardiac output through water and salt retention, peripheral vasoconstriction, increased cardiac contractility, and myocardial hypertrophy [4].

However, RAAS also determines the progression of heart failure, causing diastolic and subsequently systolic dysfunction and progression to the final stage, namely, that of congestive heart failure. This occurs as a result of cardiac remodelling which involves increased myocardial stiffness secondary to the process of hypertrophy and myocardial fibrosis. The activation of the mediators of inflammation and oxidative stress by RAAS causes endothelial dysfunction and consequently altered microcirculation, subendocardial ischemia, and exacerbation of myocardial fibrosis and heart stiffness [5, 6].

Angiotensin II (Ang II) is the main molecule in RAAS causing vasoconstriction, myocardial hypertrophy, and increased oxidative stress [7].

ACE2 opposes the effects of Ang II by catabolizing and transforming it into Ang 1-7 which has a protective role in the cardiovascular system [8]. The serum ACE2 activity has increased values in patients with heart failure and systolic dysfunction, and in some studies, it is considered a biomarker for the identification of patients with a low ejection fraction heart failure [9].

The apelin/APJ system is also an endogenous counterregulator of RAAS by antagonizing the Ang II activity [10].

Apelin is a peptide encoded by the APLN gene, located on the long arm of the X chromosome. It is synthesized as a pre-pro-apelin consisting of 77 amino acids. Apelin is subsequently cleaved into smaller C-terminal fragments with biological activity (apelin-13, apelin-16, apelin-17, apelin-19, and apelin-36) [11]. From a 77-amino acid protein, the angiotensin-converting enzyme splits apelin-13, which is the most forceful peptide and the shortest of all the fragments derived from pre-pro-apelin. During the development of heart failure, the apelin-13 plasmatic level decreases progressively, following its rise in the initial disease phases [12, 13].

There is evidence that ACE2 is the enzyme that fragments apelin by removing the C-terminus, the main area through which it exerts its effect on the APJ receptor [12, 14, 15]. In patients with a low ejection fraction heart failure, serum activity of ACE2 has elevated values, and apelin appears to have a positive regulatory role in ACE2 in these patients. The relationship between apelin and ACE2 is not fully known, requiring further clinical trials [16].

Natriuretic peptides (BNP and NT-proBNP) are now widely used in the diagnosis and follow-up of patients with heart failure. Natriuretic peptides are protein molecules that are secreted by the ventricular muscles in response to volume or pressure overload. These peptides act as a counterregulator of RAAS causing natriuresis, diuresis, vasodilation, and improved myocardial relaxation. Their role is similar in predicting the unfavourable evolution of patients with heart failure [17].

Interleukin 17 (IL-17) is a proinflammatory cytokine, which is synthesized by macrophages, helper T cells, dendritic cells, and natural killer cells. Studies in mouse models have shown that IL-17 is involved in cardiac remodelling following myocarditis and in its progression to dilative cardiomyopathy. IL-17 causes cardiac fibrosis by producing several cytokines with a role in the recruitment of neutrophils and monocytes to the inflammation site, in the activation of the matrix metalloproteinases, and in the apoptosis of heart cells [18].

Oxidative stress has high values in heart failure. 8-Iso-PGF2*α* (synonymous with 15-F2t-IsoP) belongs to the class of F2-isoprostans. They are isomers of prostaglandin PGF2*α* and are accurate markers of oxidative stress in vivo due to their stability (they are not influenced by self-oxidation and are relatively easy to measure). 8-Iso-PGF2*α* is involved in cardiac remodelling causing proliferation of fibroblasts and cardiomyocytes [19].

The aim of the study was to determine the prognostic role of biomarkers in the evolution of patients with heart failure and the possible interrelationships between biomarkers and heart remodelling. These biomarkers are representative of different pathogenetic pathways involved in the progression of heart failure.

## 2. Materials and Method

### 2.1. Study Population

Our research is a prospective observational cohort single-centre study on 53 patients diagnosed with heart failure with reduced ejection fraction, hospitalized in the Cardiology Ward of the Emergency County Hospital during a 12-month period of time, with the approval of the Bio-ethics Commission of “Iuliu Hațieganu” University of Medicine and Pharmacy, Cluj-Napoca (No. 377/03NOV2014).

The diagnosis was based on clinical evaluation, biomarker determination, and echocardiography. A control group was used; it was formed of 13 patients with arterial hypertension, which was well controlled with betablockers and/or ACEI, according to the European Society of Cardiology guidelines. The inclusion criteria were heart failure with reduced ejection fraction due to dilative cardiomyopathy or ischemic heart disease. We excluded the patients with significant valvular diseases other than secondary mitral and tricuspid regurgitation, acute myocardial ischemia, diabetes mellitus, severe renal impairment (creatinine clearance < 35 mL/min), untreated arterial hypertension, neoplasia (either current or in their history) (which had been treated surgically or by radio- and/or chemotherapy), acute focal infections (over the previous 6 months) or chronic focal infections, and collagen diseases (with or without immunosuppressive treatment).

All patients included in the study gave their written consent regarding their participation, and the presented study was approved by the Bio-ethics Commission of “Iuliu Hațieganu” University of Medicine and Pharmacy, Cluj-Napoca [20].

### 2.2. Study Protocol

All the patients were evaluated twice, once during the first admission and the second time after a period of 6-12 months. On both evaluations, the same parameters were recorded, namely, the clinical, biological, and echocardiographic ones. On admission, the NYHA class was recorded.

For biological investigation purposes, blood was collected via venous puncture, under sterile conditions and with minimal venous compression. Biological dosing was performed with the aid of the KONELAB 30 I analyser, and it was as follows: blood sugar, HDL-cholesterol, LDL-cholesterol, uric acid, triglycerides, C reactive protein, creatinine clearance (mL/min), and corrected creatinine clearance (mL/min/1.73 m^2^). A sample of serum was stored at -80°C, from which the determinations of apelin-13, ACE2, IL-17, and NT-proBNP were made, using ELISA commercial kits. Urine samples were stored in Eppendorf vials at -80°C after adding 10 *μ*L of hydroxytoluene butylate per each mL of urine as antioxidant. The 8-iso-PGF2*α* was assessed by the immunoenzymatic method using a commercially available kit from Cayman Chemical Company, Ann Arbor, Michigan, USA, with IC50 = 10 pg/mL and a detection threshold of 2.7 pg/mL. 8-Iso-PGF2*α* levels were adjusted to the urine creatinine level assessed from the same urine sample using the reaction between urinary creatinine and alkaline picrate (intratest and intertest variability coefficients of 2 and 4%, respectively).

The patients underwent an ECG on admission and daily during their hospitalization, then also on reevaluation.

An echocardiography was performed for each patient using a 2-5 MHz transducer on a Siemens Acuson X300 ultrasound machine to evaluate left ventricle (LV) structural and performance parameters.

The echocardiography was used to track the dimensions of the left ventricle and its systolic and diastolic functions, the presence of wall motion abnormalities, significant valvulopathies (valvular stenosis or failure), and a pericardial collection. The left ventricular ejection fraction was calculated using Simpson's rule in which normal adult levels were considered to be 52-78%. All parameters were calculated as the arithmetic mean of 3 determinations, in the case of patients experiencing atrial fibrillation. The relative wall thickness (RWT) was calculated after measurements of end-diastolic volume and cardiac wall thickness, a value > 0.42 meaning eccentric hypertrophy [21, 22].

The evolution was considered unfavourable when the patients presented complications during hospitalization (arrhythmias, left ventricular failure, and inadequate response to depletive therapy), were readmitted for cardiac decompensation, or died.

### 2.3. Statistical Analysis

The statistical analysis was performed with the IBM SPSS 26 software package. The data were analysed descriptively and prescriptively. Because the data were not normally distributed, nonparametric tests were performed.

The Kruskal-Wallis test was used if statistically significant differences between patients in more than two categories were detected. A Dunn-Bonferroni post hoc test was performed to determine the categories between which the difference was significant. The significance was adjusted by the sample size.

The Spearman correlation was used to assess if there were significant associations between variables as the variables were measured on a continuous scale but were not normally distributed.

The receiver-operating characteristic (ROC) curve was used to assess the accuracy of tests using the considered predicting variables. The predictive ability of each variable was determined by analysing the area under the curve (AUC).

A binary logistic regression is a technique used to determine the impact of multiple independent variables on one dichotomous dependent variable. It uses the maximum likelihood method to correctly predict the category of the outcome variable [23].

## 3. Results

Baseline clinical and demographic characteristics of patients (*n* = 53) and for the control group (*n* = 13) are shown in Table 1.

The unfavourable evolution was recorded for 14 (24.52%) of the 53 patients followed up over a 12-month period.

An analysis of the ROC curve was performed to determine the ability to predict the indicators: apelin-13, ACE2, IL-17, hsCRP, 8-iso-PGF2*α*, NT-proBNP, and the ejection fraction. Analysing the value of the area under the curve (AUC), it was observed that only ACE2, apelin-13, NT-proBNP, and hsCRP presented statistically significant values indicating that they have an ability to predict evolution (Table 2).

**Figure 1:**
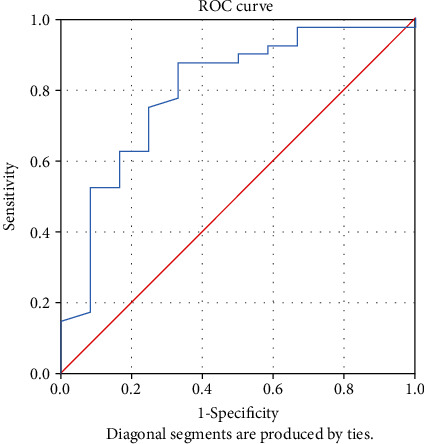
ROC curve for variable ACE2.

The ROC was used to identify the optimal cut-off value for variable ACE2. The optimal cut-off value for predicting the patient's evolution was identified at 4000.75 pg/mL with a sensitivity of 87.5% and a specificity of 66.7% (*p* = 0.002). The area under the curve (AUC) was 0.796 (95% CI 0.644–0.947) indicating that the variable can be used to predict the evolution of the patient (Figure 1).

**Figure 2:**
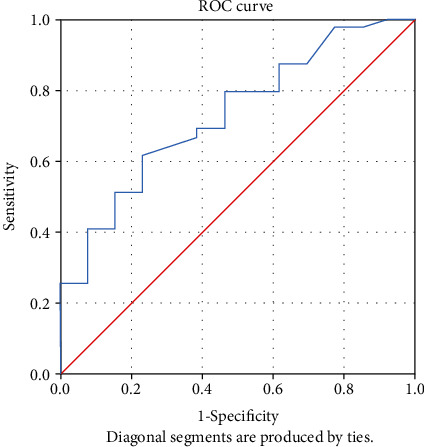
ROC curve for variable apelin-13.

The ROC was used to identify the optimal cut-off value for variable apelin-13. The optimal cut-off value for predicting the patient's evolution was identified at 402.5 pg/mL with a sensitivity of 61.5% and a specificity of 76.9% (*p* = 0.013). The AUC was 0.731 (95% CI 0.581–0.881) (Figure 2).

**Figure 3:**
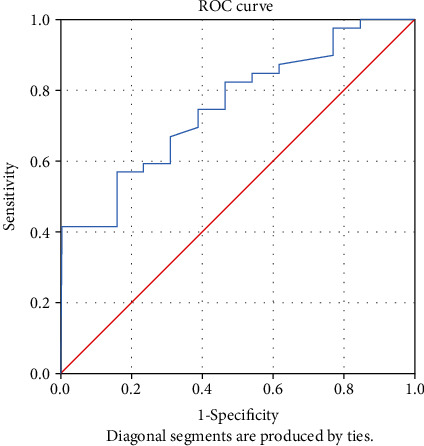
ROC curve for variable NT-proBNP.

The ROC was used to identify the optimal cut-off value for variable NT-proBNP. The optimal cut-off value for predicting the patient's evolution was identified at 911 (pg/mL) with a sensitivity of 41% and a specificity of 100% (*p* = 0.006). The AUC was 0.754 (95% CI 0.615–0.894) (Figure 3).

**Figure 4:**
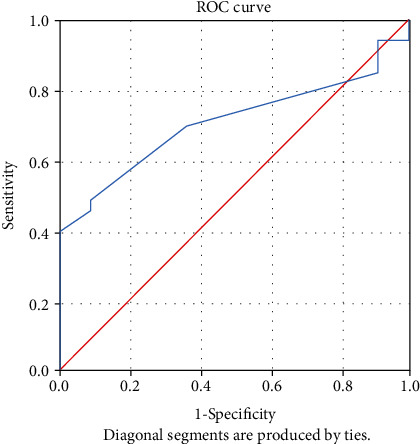
ROC curve for variable CRP.

The ROC was used to identify the optimal cut-off value for variable CRP. The optimal cut-off value for predicting the patient's evolution was identified at 0.485 (mg/dL) with a sensitivity of 48.5% and a specificity of 90.9% (*p* = 0.042). The AUC was 0.707 (95% CI 0.556–0.857) (Figure 4).

### 3.1. Multivariate Analysis

The variables that had significant predicative power were used in a multivariate analysis. The variable NT-proBNP was removed from the analysis because it was highly correlated with the apelin-13 variable.

A binary logistic regression was performed to ascertain the effect of ACE2, apelin-13, and CRP on the evolution of the patient. The logistic regression model was statistically significant, *χ*^2^(3) = 22.090, *p* < 0.001. The model explained 50.7% (Nagelkerke *R*^2^) of the variance in the evolution of the patient and correctly classified 84.9% of the patients. An increase in ACE2 values is associated with a slight increase in the likelihood of patients evolving unfavourably. Decreasing values of apelin-13 were also associated with a slight negative evolution of the patients. The regression coefficients (*B*) and their associated significance (Sig.) can be found in Table 3.

An analysis was performed using the Spearman correlation to test whether there is an association between the indicators ACE2, IL-17, apelin-13, NT-proBNP, the ejection fraction, and 8-iso-PGF2*α*. A strong negative correlation was identified between the serum values of apelin-13 and NT-proBNP (rs = −0.822, *p* < 0.01).

A Kruskal-Wallis test was performed to determine whether there was a significant difference between patients in different NYHA classes of apelin-13. A significant difference was found between patients from different NYHA classes (*H* (2) = 13,497, *p* = 0.001). Applying a Dunn-Bonferroni post hoc test, it was observed that NYHA IV class patients had significantly lower apelin-13 values than those in class II (*Z* = 3.457, *p* = 0.002) and class III (*Z* = 3.322, *p* = 0.003).

A Kruskal-Wallis test was performed to determine whether there was a significant difference between patients in different NYHA classes of hsCRP values. A significant difference was found between patients from different NYHA classes (*H* (2) = 15.163, *p* = 0.001). Applying a Dunn-Bonferroni post hoc test, it was observed that NYHA class IV patients had significantly higher hsCRP values than class II patients (*Z* = −3.751, *p* < 0.001).

An analysis was performed using the Spearman correlation to test whether there was an association between the following indicators: ACE2, IL-17, apelin-13, NT-proBNP, the ejection fraction, and 8-iso-PGF2*α* on the one hand and, on the other hand, whether there was an association between the end-diastolic LV diameter, the end-systolic LV diameter, the end-diastolic LV volume, the end-systolic LV volume, LV mass, and the relative parietal thickness regardless of the NYHA class in which the patients find themselves.

All heart failure patients had high values of LV mass (median 291.5 g; IQR = 263–344.75), relatively stable during the follow-up (314 median, IQR = 263–372 on reevaluation, *p* = 0.877).

A significant negative correlation was found between end-systolic volume and apelin-13 (rs = −0.309, *p* = 0.032).

In the case of NYHA class IV, a significant positive correlation was found between the serum value of apelin-13 and the RWT (rs = 0.807, *p* = 0.015).

In NYHA class II patients, a negative correlation was identified between 8-iso-PGF2*α* and the RWT (rs = −0.461, *p* = 0.020).

In the case of NYHA class III, significant negative correlations were found between the ejection fraction and the end-systolic diameter (rs = −0.487, *p* = 0.034).

## 4. Discussion

Our study showed that in congestive heart failure, biomarkers from different molecular pathways can be concurrent for an accurate evaluation of the prognosis and can be used to identify the patients at risk of an unfavourable outcome.

In our study, the levels of ACE2 higher than 4000.75 pg/mL had a high predictability capacity for an unfavourable outcome, with an AUC of 0.796, a sensitivity of 87.5%, and a specificity of 66.7% (*p* = 0.002). Also, a level of apelin-13 lower than 402.5 pg/mL had a high predictability capacity for an unfavourable outcome, with an AUC of 0.731, a sensitivity of 61.5%, and a specificity of 76.9% (*p* = 0.013). These results confirmed the importance of these biomarkers for an accurate evaluation of the prognosis of a patient with congestive heart failure. This dynamics is in concordance to other reports, both experimental and clinical studies [24, 25], especially with the new observation of higher levels of plasma ACE2 being a risk factor for cardiovascular disease and death [26].

The apelin system is in direct interconnection with RAAS, and the overstimulation of this system induces both the reduced synthesis of apelin and its increased degradation by ACE2 enzyme [12, 24]. The relationship between the apelin system and the ACE2 was proved previously, but their dynamics in the different stages of heart failure was not completely described and understood [3], and this study tried to bring more light into this problem.

The role of ACE2 enzyme is complex and powerful and is yet to be discovered in its entirety. ACE2 enzyme is the main counteraction of RAAS by degrading the angiotensin II (Ang II) and angiotensin I (Ang I) to angiotensin 1-7 (Ang 1-7) and angiotensin (Ang 1-9), respectively, two nonaggressive peptides on the failing myocardium, protective for the cardiovascular system, in contrast to the proinflammatory and profibrotic Ang I and II [27–29]. In experimental models, the ACE2 expression is downregulated in apelin-knockout mice and is upregulated by administering exogenous apelin [16]. But also, ACE2 inactivates apelin, and these two divergent actions need further investigation and clarification [25, 27, 30]. In our study groups, we included patients and controls that were already under ACEI treatment, in order to equalize the differences that may come from this therapy on the plasma levels of these sensible peptides. Although apelin-13 and ACE2 are parts of the cardioprotective molecular pathways and both in opposition to RAAS, their dynamics proved to be different.

These observations are in line with the new reports about the increased levels of ACE2 in advanced cardiovascular disease and with the negative prognostic value of high levels of ACE2, both for cardiovascular disease and death of all causes [26].

The relationship between apelin and NT-proBNP was analysed in several studies of heart failure patients, as is the research of Chandrasekaran et al. or Miettinen et al. [31–33]. Although the previous results were discordant, in our study, the relationship between apelin-13 and NT-proBNP was confirmed through the strong, negative correlation between these two biomarkers in the patient group (rs = −0.822, *p* < 0.01). Basically, as the heart failure aggravates and the congestion increases, the level of NT-proBNP rises and those of apelin-13 lowers [15].

The patients with NYHA IV heart failure and lower ejection fraction had significantly higher levels of NT-proBNP and ACE2 and significantly lower levels of apelin-13 compared to the patients with NYHA II heart failure. In the multivariate analysis, the decrease of apelin-13 and the increase in ACE2 were associated with a slight but significant increase of adverse events.

In our analysis, we observed a significant negative correlation between left ventricle end-systolic volume and the level of apelin-13 (rs = −0.309, *p* = 0.032). The relative wall thickness has a negative correlation with the level of urinary 8-*iso-*PGF2*α* in the group of NYHA II patients (rs = −0.461, *p* = 0.020) and a positive correlation with the apelin-13 level in the group of NYHA IV patients (rs = 0.807, *p* = 0.015). The failing heart undergoes a remodelling process that leads to its dilation, with or without associated LV hypertrophy, and to the alteration of the global geometry of the cavity. LV diameters increase, and the cavity becomes spherical, changing the distribution of the cavitary and wall stress, raising the oxygen demand of the myocardium. All of our heart failure patients had very high LV mass, so the RWT showed concentric (RWT > 0.42) or eccentric (RWT ≤ 0.42) LV hypertrophy. The higher values of RWT (concentric LV hypertrophy) were correlated with higher values of apelin-13 and lower levels of urinary 8-*iso-*PGF2*α*, meaning the eccentric hypertrophy is associated with opposites values of these biomarkers. In our observation, the lower levels of apelin-13 and higher levels of oxidative stress are associated with higher NT-proBNP levels, more severe heart failure, and unfavourable outcome during the follow-up.

The heart failure is a chronic, constantly progressive disease, which promotes inflammation and also is aggravated by inflammation. It induces inflammation inside the myocardium due to the wall stress, hemodynamic factors, and the overstimulation of the RAAS. The patients with more severe heart failure (NYHA class IV) had higher levels of CRP and lower levels of apelin-13 than the patients in NYHA class II (*Z* = −3.751, *p* < 0.001 and *Z* = 3.457, *p* = 0.002, respectively), and the level of apelin was significantly lower in NYHA class IV than in NYHA class III also (*Z* = 3.322, *p* = 0.003). These observations are concordant to other studies that showed higher levels of CRP in heart failure patients [34] and raise questions about a potential causal relationship between the higher inflammation, apelin decrease, and the unfavourable prognosis in the late stages of heart failure.

The cardiac remodelling by itself is an independent factor for the progression of heart failure, leading to congestion; the biomarkers studied here are parts of a molecular network with strong interconnections. The overstimulation of RAAS is a constant aggression for the failing myocardium that apelin and ACE2 systems are trying to overcome, the alteration of their serum levels, as described above, being the signs for the adverse outcome that is to come.

## 5. Conclusions

The serum values of ACE2 and apelin-13 correlate with the unfavourable outcome in patients with reduced ejection fraction heart failure and may be useful biomarkers in determining the prognosis of these patients.

## Figures and Tables

**Table 1:** The baseline characteristics of patients and controls.

		Patients (*n* = 53)	Controls (*n* = 13)	*p* value
Age (years)		67.94 (11.81)	55.38 (10.69)	<0.001
BMI (kg/m^2^)		26.2 (24.2-30)	29.38 (26.6-30.37)	0.273
Sex (female)		15 (28.3)	9 (69.23)	0.01
Cigarette smoking		15 (28.3)	4 (30.77)	1
Alcohol drinking		10 (18.87)	2 (15.38)	1
Heart failure	Acute (de novo) HF	14 (26.42)	—	—
	Chronic compensated HF	19 (35.85)		
	Chronic decompensated HF	20 (37.74)		
NYHA class	II	25 (47.17)	—	—
	III	19 (35.85)		
	IV	9 (16.98)		
Medication	Betablocker	2 (3.77)	1 (7.69)	0.185
	ACE inhibitor	11 (20.75)	0 (0)	
	Betablocker+ACE inhibitor	28 (52.83)	7 (53.85)	
	None	12 (22.64)	5 (38.46)	
LV end-diastolic volume (mL)		146 (120-190)	—	—
LV mass (g/m^2^)		162.5 (41)	90.6 (17)	<0.001
Ejection fraction (%)		25 (20-35)	55 (55-60)	<0.001
NT-proBNP (pg/mL)		1241 (875-1531)	254 (100-334)	<0.001
Apelin-13 (pg/mL)		495 (275-845)	515 (402-1005)	0.223
ACE2 (pg/mL)		3123 (2780–3467)	3106 (2543–3669)	0.966
IL-17 (pg/mL)		1.26 (1.045–1.615)	1.64 (0.99–2.30)	0.268
8-Iso-PGF2*α* (pg/*μ*mol creatinine vs)		267.32 (299.82-636.5)	19.82 (13.35-22.5)	*p* < 0.001
Uric acid (mg/dL)		7.5 (6.5-8.9)	4.6 (3.8-5.2)	<0.001
Total cholesterol (mg/dL)		155 (140-174)	212 (181-219)	<0.001
LDL-cholesterol (mg/dL)		93 (72-106)	135 (109-157)	<0.001
HDL-cholesterol (mg/dL)		41 (34-48)	50 (41-62)	0.047
Triglycerides (mg/dL)		108 (87-144)	89 (69-112)	0.116
C reactive protein (mg/dL)		0.5 (0.4-0.8)	0.5 (0.3-0.5)	0.142
Creatinine clearance (mL/min)		72.73 (32.87)	103.89 (23.54)	0.002
Corrected creatinine clearance (mL/min/1.73 m^2^)		65.65 (26.08)	97.54 (19.92)	<0.001

BMI: body mass index; HF: heart failure; ACE inhibitor: angiotensin-converting enzyme inhibitor; LV: left ventricle; LDL: low-density lipoprotein; HDL: high-density lipoprotein. Categorical data are presented as number (percentage), normally distributed continuous data are presented as mean (standard deviation), and those not normally distributed are presented as median and interquartile range.

**Table 2:** AUC and *p* values for the studied parameters.

	AUC	*p*
ACE2	0.796	0.002
Apelin-13	0.731	0.013
Ejection fraction	0.645	0.119
IL-17	0.472	0.774
8-Iso-PGF2*α*	0.421	0.411
NT-proBNP	0.754	0.006
CRP	0.707	0.042

**Table 3:** Regression model coefficients for predicting patient evolution.

Variable	*B*	S.E.	Wald	df	Sig.	Exp(*B*)	95% CI for Exp(*B*)	
							Lower	Upper
ACE2	0.001	0	8.805	1	0.003	1.001	1	1.002
Apelin-13	-0.005	0.003	4.234	1	0.04	0.995	0.99	1
CRP	0.048	0.162	0.087	1	0.768	1.049	0.763	1.442
Constant	-3.209	1.551	4.282	1	0.039	0.04		

## Data Availability

The clinical data used to support the findings of this study are included within the article.
